# Real-time gated proton therapy: Introducing clinical workflow and failure modes and effects analysis (FMEA)^☆^

**DOI:** 10.1016/j.tipsro.2025.100311

**Published:** 2025-05-02

**Authors:** Wei Yang Calvin Koh, Hong Qi Tan, Kah Seng Lew, Wan Ting Alice Kor, Nur Atiqah Binte Samsuri, Jason Wei Siang Chan, Clifford Ghee Ann Chua, James Kuan Huei Lee, Andrew Wibawa, Zubin Master, Sung Yong Park

**Affiliations:** a Division of Radiation Oncology, National Cancer Centre Singapore, Singapore; b Oncology Academic Clinical Programme, Duke-NUS Medical School, Singapore; c Department of Physics, National University Singapore, Singapore; d School of Physical and Mathematical Sciences, Nanyang Technological University Singapore, Singapore

## Abstract

**Introduction:**

Real-Time Gated Proton Therapy (RGPT) has been used in proton therapy to mitigate respiratory motion challenges. This study presents the first comprehensive description of the RGPT clinical workflow at the National Cancer Centre Singapore (NCCS) and introduces a novel Failure Modes and Effects Analysis (FMEA) for RGPT.

**Methods:**

We detail the workflow for prostate and liver/lung proton treatments. The FMEA, following AAPM TG-100 guidelines, evaluated potential failure modes based on occurrence, detectability, and severity. This comprehensive risk assessment approach allows for the establishment of robust Quality Assurance (QA) protocols, enhancing treatment safety and efficacy. The FMEA was performed by two disciplinary groups, namely the medical physicists and radiation therapists. Intraclass Correlation Coefficient (ICC) is used as the metric to assess the consistency of ratings among participants.

**Results:**

Since implementation, 15 patients (13 prostate, 2 liver) have been treated with RGPT. The study identified 96 potential failure modes, with 47 evaluated by two disciplines. Inter-rater concordance analysis revealed strong agreement within one group, while combined groups showed lower consistency, highlighting potential differences in risk perception between disciplines. Notably, severity ratings demonstrated better correlations among raters compared to other categories, suggesting a shared understanding of the potential impact of failures.

**Conclusion:**

This study provides valuable insights for institutions implementing RGPT, potentially enhancing treatment workflow, patient safety, and QA procedures. The detailed workflow description and FMEA results offer a foundation for standardizing RGPT practices and prioritizing risk mitigation strategies. Future research should focus on multi-institutional collaborations to further refine RGPT protocols and risk assessments.

## Highlights

• Proton Therapy Pencil Beam Scanning.
• FMEA to enhance treatment workflow, patient safety and quality assurance procedures.
• Clinical implementation and risk assessment of Real-Time Gated Proton Therapy.
• Establish Real-Time Gated Proton Therapy Clinical Workflow and Quality Assurance.
• Patient Safety.

## Introduction

Proton beam therapy offers significant dosimetric advantages over conventional X-ray therapy by potentially reducing normal tissue toxicities while achieving tumor coverage. Pencil beam scanning (PBS) is a technique which enhances this precision by delivering multiple proton spots to the planned location [1,2]. However, the efficacy of PBS is challenged by respiratory motion, which can cause dose degradation through the interplay effect, leading to hot and cold spots within the tumor [[3], [4], [5]].

A Real Time-Gated Proton Therapy (RGPT) in PBS proton beam therapy was developed from the collaboration between Hokkaido University Hospital and Hitachi Ltd to address these challenges. RGPT gates the proton beam by tracking the implanted fiducial markers. These fiducial markers act as a surrogate for tumor position and the proton beam is only irradiated when the marker is in the planned position, thereby improving treatment accuracy and reducing doses to normal tissues.

The National Cancer Centre Singapore (NCCS) and The Johns Hopkins University School of Medicine have recently published their work on the commissioning of their RGPT system [5,6]. Currently, there are no publications that share the clinical workflow (prostate, liver and lung treatment sites) of RGPT in PBS proton therapy. This paper provides an in-depth description of the RGPT clinical workflow at NCCS, detailing the procedure from fiducial marker insertion to patient treatment. The RGPT in NCCS is unique, featuring the smallest source to imager distance (SID) in the world for enhanced image quality and marker visibility. This configuration optimizes the accuracy and effectiveness of RGPT, contributing to better treatment outcomes.

NCCS has previously reported on the commissioning and device-specific quality assurance in RGPT [5]. This study aims to present the clinical workflow and the failure modes and effect analysis (FMEA) of the RGPT treatment process, identifying potential failure modes and their effects on treatment. FMEA has been widely utilized in healthcare and radiotherapy settings to improve patient safety and treatment efficacy [[7], [8], [9], [10]]. This methodology has been applied to evaluate and implement emerging technologies in healthcare [11,12]. It has also been used to refine clinical protocols and operational workflows to reduce risks [13,14].

FMEA serves as an essential instrument for decision-making, enabling the establishment of preventive measures and detection mechanisms for potential failures that could compromise patient care [15]. While assembling an FMEA team can be logistically challenging [16], a recent study suggests that integrating both prospective and retrospective approaches can enhance safety protocols, allowing for real-time identification of previously unforeseen failure modes [17].

Our study builds upon this body of knowledge by applying FMEA specifically to the RGPT treatment process, an area that has not been previously explored in the literature. Following the AAPM TG-100 Report guideline, each failure mode is scored and evaluated based on 1) the probability of occurrence, 2) the detectability of the failure and 3) the severity if the failure goes undetected. By sharing these comprehensive procedures, we believe that this work could benefit new centres that are interested in implementing RGPT in their clinic. The detailed clinical workflow and FMEA presented in this study could serve as a guideline for other institutions, contributing to the advancement of precision cancer treatment and improving patient safety and treatment efficacy.

## Methods

**Overview of NCCS RGPT system and workflow**

At NCCS, the Hitachi Probeat Proton therapy system is a synchrotron-based accelerator which uses a pencil beam spot scanning delivery method [5,18,19]. The machine can deliver 98 energies ranging from 70.2 MeV to 228.7 MeV and has an in-air 1σ spot size of 1.94 mm to 5.16 mm at the isocentre. There are four full rotating gantries and each treatment room is equipped with two sets of X-ray sources and detectors, all capable of performing RGPT. This RGPT system, with a SID of 1.6 m, is the smallest in the world as compared to other Hitachi centres using RGPT. This unique configuration of the RGPT system was designed to achieve better image quality and thus, better marker visibility and RGPT performance.

This study reports the process map for RGPT treatment and the clinical workflow for each treatment site treated using RGPT (prostate, lung-and-liver). FMEA was then applied to all the subprocesses involved, to ensure the safety of patients undergoing RGPT treatment.

**Clinical workflow for prostate RGPT**

At our centre, only the patients receiving irradiation to the primary *prostate gland* are treated with RGPT. Patients with pelvic lymph node irradiation are excluded from RGPT. Upon patient selection, they will be scheduled for fiducial marker insertion prior to the RGPT assessment and CT Imaging. Instructions on the type of markers and the marker placement are provided to the urologists. Some criteria are strictly required to be adhered to such as having at least two markers inserted in the patient within 10 cm from the planned isocenter (usually placed at the centre of the prostate gland). The position of the marker insertion is limited by the field of view of our imaging system and three markers are recommended to circumvent marker migration occurrence and to allow for accurate rotation correction during the initial patient setup using cone-beam computed tomography. In the clinical workflow of RGPT treatment, there are two components: an *Assessment Workflow* and a *Treatment Workflow*.

***Assessment Workflow***

An RGPT *assessment* workflow was established to ensure the feasibility of performing RGPT for the intended patient. The patient is first placed on the couch and fluoroscopy images are taken at gantry angles 315° and 270°. The initial fluoroscopy settings of 125 kVp, 50 mA and 1 PPS (Pulse Per Second) are used to assess the visibility and the tracking stability of all fiducial markers. These settings can be modified depending on the matching score of the markers [5]. Thereafter the ideal x-ray settings and the marker with the highest matching score will be documented for treatment reference.

***Treatment Workflow***

In the RGPT *clinical* workflow for prostate cancer treatment, patients are set up using Cone Beam CT (CBCT) imaging upon adhering to the bladder protocol. Bone match with 6 degrees of freedom (DoF) via CT-CBCT images was done with a tolerance of 1.5 cm / 3° and the shifts were applied. If the shifts exceed the tolerance, a re-setup is required. The tolerance is a re-setup uncertainty and part of the safety workflow. It serves as a cautionary measure that indicates the need for closer examination if a significant or large bone match discrepancy occurs.

**Fig. 1:**
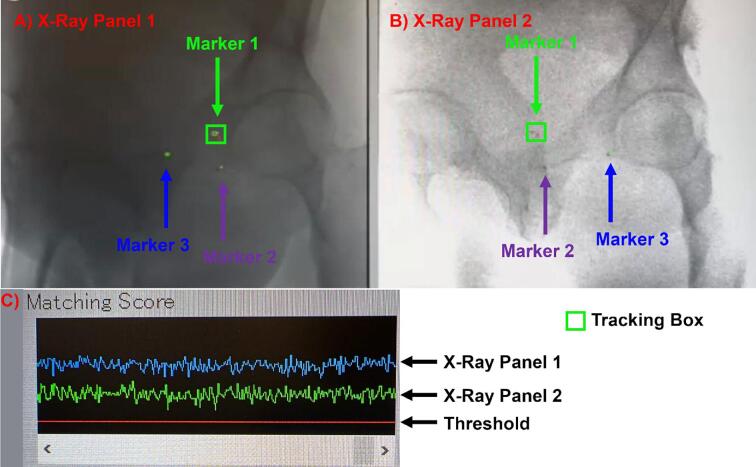
A) X-ray Panel 1. 1B) X-ray Panel 2. C) Matching score of both X-ray panels (Green − X-ray Panel 1, Blue − X-ray Panel 2) on **Marker 1**. The red line depicts the threshold of 30. (For interpretation of the references to colour in this figure legend, the reader is referred to the web version of this article.)

Prior to the start of each treatment field, a pair of fluoroscopy images is first taken and a 3 DoF marker match is performed. An RGPT tracking template is created based on the fluoroscopy images and the matching score of the marker needs to be greater than 30 as shown in Fig. 1.

The 3 DoF marker match shifts ($\Delta X_{\mathrm{marker}},\Delta Y_{\mathrm{marker}},\Delta Z_{\mathrm{marker}}$) are documented to track the total RGPT marker match shifts. To ensure that the prostate is not shifted excessively relative to the body which could compromise the target coverage, a maximum allowable marker shift tolerance is defined in our institution. The total RGPT marker shifts need to be within the tolerance level as defined in Equation (1) to continue treatment otherwise a re-setup with CBCT is warranted.

(1) $$\sum_{i=1}^{n}\Delta M_{\mathrm{marker},i}<Maximum\;allowable\;marker\;shifts$$

refers to marker shift applied in the X, Y and Z directions while n refers to the number of times RGPT shifts are applied during the treatment. The maximum allowable marker shifts follow the same concept as presented by Lee et. al. [20]. The maximum allowable marker shifts are determined by comparing the CTV D_95%_ values to clinical goals where an in-house algorithm is used to calculate the CTV D_95%_ values at specific user-defined marker shifts. The clinical goal adopted by NCCS is CTV D_95%_ > 95 % of the prescribed dose.

**Fig. 2:**
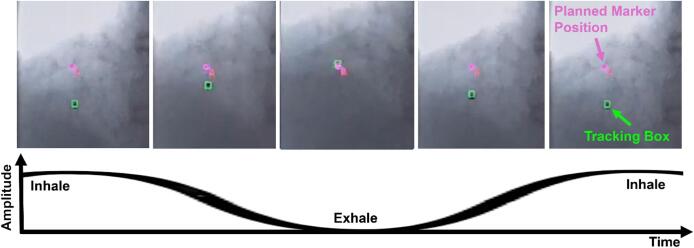
Fiducial marker motion trajectory for liver tumor. The pink marker indicates the plan position while the green box tracks the actual fiducial marker position. (For interpretation of the references to colour in this figure legend, the reader is referred to the web version of this article.)

Lastly, upon applying the RGPT marker shifts, fluoroscopy is turned on and the marker tracking starts. Once the abovementioned criteria are fulfilled, the proton irradiation will start. During the treatment, the marker tracking and the matching score are monitored to ensure that there is no loss in marker tracking or actual deviation of the markers. The loss of marker tracking refers to the tracking box (Fig. 2) rapidly sweeping the region in search of the marker until it re-establishs a stable tracking state on the marker. In the case of loss of marker tracking, recovery actions can be performed to circumvent the issue. Radiation therapists can continuously select the marker during the treatment such that the marker is being tracked correctly or the treatment irradiation can be paused to increase the kVp and mA settings of the treatment for better marker contrast and tracking. However, when the markers do deviate from the plan, this can be caused by (1) organ deformation, (2) slight patient movement or (3) larger patient movement exceeding RGPT maximum allowable shifts. For cases (1) and (2), radiation therapists can repeat the marker-matching process and start treatment thereafter. For case (3), a re-setup is required. The monitoring of the marker tracking and the matching score will be perpetual until the completion of the treatment.

**Clinical workflow for Liver/Lung RGPT**

For lung and liver lesion cases, the main difference lies in the machine-specific parameters such as x-ray settings (kV, mA and PPS) and the workflow when grappling with the periodically moving marker due to respiration motion in both the RGPT *assessment* and the *clinical* treatment.

***Assessment Workflow***

In the RGPT *assessment* session, the PPS used for fluoroscopy will be set between 15 and 30 PPS. The marker template is generated at the exhalation phase of the breathing cycle as shown in Fig. 2 to enhance treatment reproducibility and extend the dwell time for treatment [[21], [22], [23], [24], [25]]. Thereafter, the marker is then assessed for 2–3 breathing cycles for its visibility and tracking stability. The ideal marker and fluoroscopy settings are then recorded for treatment reference.

***Clinical Workflow***

In the *clinical* workflow for liver/lung cases, the marker template is created at the exhale phase of the breathing cycle with the reference fluoroscopy settings. Similar to prostate *clinical* workflow, the RGPT marker shifts are required to be within the RGPT maximum allowable shifts and the marker matching score has to be greater than 30. Before irradiation, the fluoroscopy images are taken and there should be no marker tracking loss for at least 2–3 breathing cycles. These steps are repeated for all treatment fields and with these protocols in place, the RGPT irradiation can be performed.

**Failure modes and effects analysis (FMEA)**

The FMEA on RGPT treatment was conducted with reference to the TG100 report from AAPM [26]. A process map (PM) was first created by medical physicists based on the 7 processes in the RGPT treatment workflow. The FMEA table with the rating scale as proposed by the TG100 report was generated. The risk priority number (RPN) was calculated for all failure modes as shown in Equation (2).

(2) RPN=O×S×D

RPN is defined by the product of Occurrence (O), Severity (*S*) and Detectability (*D*). The RPN threshold of 125 considered the risk acceptable and this had been applied by various previous FMEA studies in radiation oncology [10,[27], [28], [29], [30], [31], [32]]. However, Broggi et. al. pointed out that this number remains arbitrary when applied in radiation oncology and requires further investigation [32].

The FMEA analysis was separated into two groups. *Group 1* consists of three medical physicists to rate all processes according to *O*, *S* and *D*. *Group 2* consists of three radiation therapists to rate only two processes − *RGPT Feasibility Assessment for Moving Target* and *Treatment*. All staff were briefed to achieve a mutual understanding of the grading system of FMEA following the TG100 report. The results are compiled both separately and combined for analysis between disciplines.

To assess the consistency of ratings among participants within each group and between the two groups, the intraclass correlation coefficient (ICC) was employed as a metric. This statistical measure provides insight into the level of agreement among raters, helping to validate the reliability of the FMEA results and identify any significant discrepancies in risk perception between medical physicists and radiation therapists. The ICC was calculated for each of the FMEA components (*O*, *S*, *D* and *RPN*) to evaluate the degree of consistency in scoring across raters and disciplines.

## Results

**Fig. 3:**
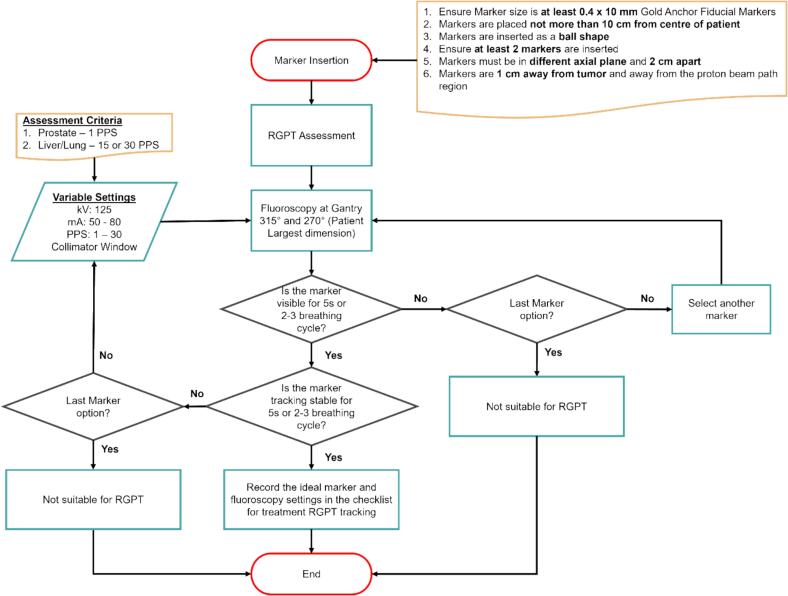
RGPT Assessment workflow for prostate, lung and liver cases.

Since the implementation of RGPT treatment, 15 patients comprising 13 prostate and 2 liver patients have been treated with RGPT. The assessment workflow is shown in Fig. 3. The primary reason for curating an RGPT assessment workflow is to ensure that the patient is a suitable candidate for RGPT treatment.

**Clinical workflow for prostate RGPT**

At NCCS, bladder protocol is practised for setup reproducibility and to reduce the dose primarily to the bowel OAR. On the first day of treatment, a large FOV CBCT is taken to ensure the contour of the patient matches with the plan. Subsequently, the weight of the patient is monitored throughout all fractions and only a small FOV CBCT is taken for better image quality on the soft tissues. Mid-treatment CBCT is taken with large FOV to verify that the whole body contour of the patient still matches with the planning CT. A re-CT and re-plan might be required if there is a significant difference between the contours.

Thereafter, marker matching is done and the RGPT marker match shifts are recorded by the radiation therapist operating the system. The total marker shifts are calculated such that it is within the maximum allowable RGPT shifts before proceeding to irradiation. If the total marker shifts exceed the maximum allowable RGPT shifts, a re-setup is required. During the treatment, the RGPT system operator has to consistently monitor for marker tracking loss or actual marker deviation. This process will repeat till all fields are treated.

**Clinical workflow for Liver/Lung RGPT**

**Fig. 4:**
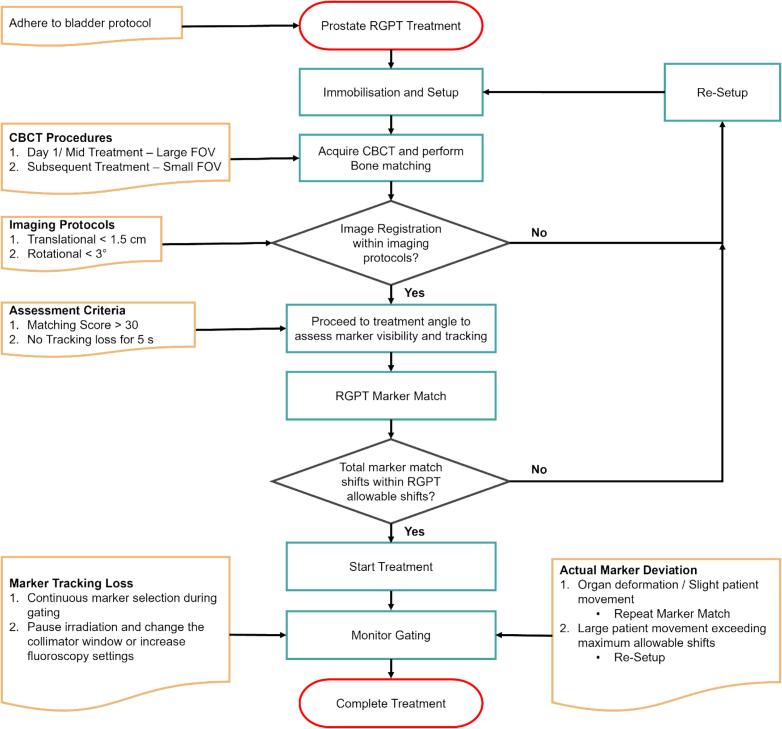
Prostate RGPT workflow.

**Fig. 5:**
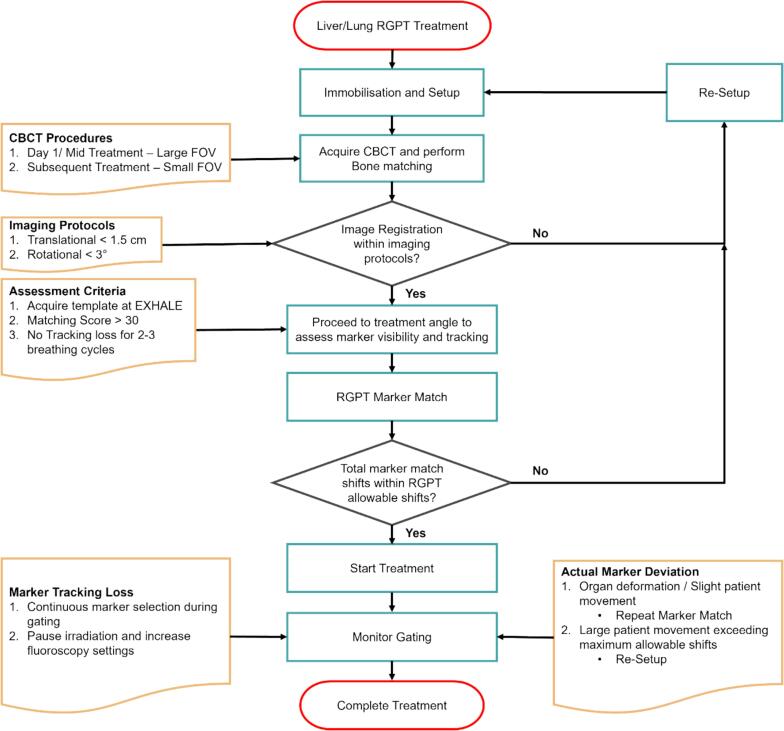
Liver/Lung RGPT workflow.

Fig. 5 shows the RGPT workflow for liver and lung cases. The main difference lies in the assessment criteria before performing the marker matching. It is important to ensure that the image for creating the RGPT tracking template is at the exhale phase of the patient. In contrast to the prostate’s workflow, 2–3 breathing cycles are required to ensure marker tracking stability for treatment of tumor under respiratory motion. The subsequent part of the workflow follows the same as the prostate workflow in Fig. 4.

**Failure modes and effects analysis (FMEA)**

The patient's RGPT treatment process comprises of 7 sections with their number of subprocesses shown in the parenthesis.

1. Fiducial Insertion (6)
2. RGPT Feasibility Assessment for Moving Target (11)
3. Computed Tomography (CT) Simulation (13)
4. Treatment Planning (16)
5. Physics Plan Checks (9)
6. Treatment (36)
7. Re-CT and Replanning (5)

For fiducial insertion, the oncologist will provide the surgeon with information on which type of gold fiducial marker to use and where it should be inserted. The standard fiducial marker used for RGPT treatment is a 0.4 x 10 mm Gold Anchor^TM^ fiducial marker (Naslund Medical AB, Huddinge, Sweden). Thereafter, the patient is scheduled for an RGPT assessment to ensure the feasibility of using RGPT for moving targets (Liver/Lung) treatment. The CT simulation will be scheduled on the same day as the RGPT assessment. Upon the confirmation of treatment with RGPT, treatment planning will be done by the dosimetrist. The treatment plan will be reviewed by the radiation oncologist and subsequently, the physicist will perform the plan checks and quality assurance checks. The treatment will then be initiated. Re-CT and re-planning will be case-specific and performed when required.

**Fig. 6:**
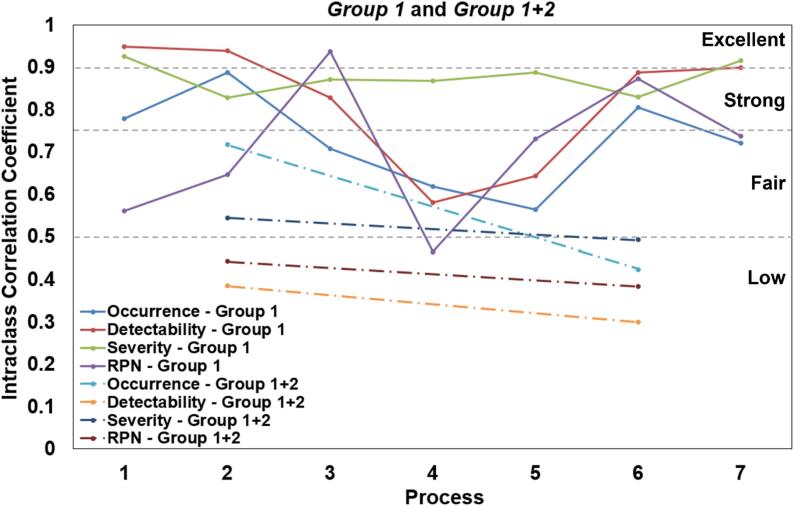
Intraclass correlation coefficient (ICC) of O, D, S, RPN for *Group 1*(solid line) and *Group 1 + 2* (dot-dashed line). ICC is only defined for Processes 2 and 6 since *Group 2* only rated these processes. The dashed line (grey) provides the range of agreement based on the ICC value where low agreement − 0 to 0.5, fair agreement – 0.5 to 0.75, strong agreement – 0.75 to 0.9 and excellent agreement − >0.9.

A total of 96 failure modes were then rated by members of *Group 1* and 47 failure modes (a subset of 96 failure modes) by both *Group 1* and *Group 2* members. Inter-rater concordance is used to assess the agreement between different raters in each category *O*, *D*, *S* and *RPN*. Fig. 6 shows the ICC calculated for each component of the FMEA across seven processes, comparing assessments made by medical physicists (*Group 1* and a combined group including radiation therapists (*Group 1 + 2*).

For *Group 1*, the ICC values demonstrated considerable variability across processes and FMEA components. Occurrence ratings showed a range from fair to excellent agreement (ICC: 0.56–––0.89), with the highest consistency observed in Process 2 and the lowest in Process 5. Detectability ratings exhibited a wider range, from fair to excellent agreement (ICC: 0.58–––0.95), peaking in Process 1 and dropping to its lowest in Process 4. Severity ratings maintained relatively stable excellent agreement across all processes (ICC: 0.83–––0.95), indicating strong consensus among medical physicists on this component. The Risk Priority Number (RPN) showed the most variability, ranging from fair to excellent agreement (ICC: 0.46–––0.95), with Process 3 showing the highest consistency and Process 4 the lowest.

When comparing *Group 1* to the combined *Group 1 + 2* (which included radiation therapists' assessments for Processes 2 and 6), notable differences emerged. For Process 2, the inclusion of radiation therapists resulted in lower ICC values across all components, most markedly in detectability (from 0.95 to 0.39) and RPN (from 0.64 to 0.44). This trend was less pronounced but still evident in Process 6, where the combined group showed lower ICC values, particularly in occurrence (from 0.80 to 0.46) and detectability (from 0.89 to 0.30).

These findings suggest that while medical physicists demonstrated generally strong internal consistency in their FMEA assessments, the inclusion of radiation therapists introduced greater variability, particularly in judging detectability and occurrence of failure modes. The consistently high agreement on severity ratings among medical physicists contrasts with the more variable assessments of other FMEA components, highlighting potential areas for focused training or standardization in the FMEA process.

Table 1, Table 2 show the top ten RPN failure modes based on average *O*, *D*, *S* and *RPN* scores for *Group 1* and *Group 1 + 2*. The top ten average RPN failure modes for *Group 1* stem from *Plan Checks*, *Treatment* and *re-CT and Replanning* processes. The top failure mode arises from the *re-CT and Replanning* process where the CBCT is incorrectly registered to the Planning CT images, leading to wrong dose evaluation and clinical decisions made for the patient. 3 out of 10 of the failure modes occur during the physics plan checks when determining the maximum allowable RGPT shifts for the patient. 6 out of 10 of the failure modes arise from the *Treatment* process. As *Group 1 + 2* only rated 2 processes, it is not surprising that the treatment process contributed to all of the top ten failure modes. The consensus among all raters for the highest *RPN* failure mode is caused by the irregular breathing cycle of the patient with an average *RPN* score of 142.

**Table 1:** *Group 1* top ten FMEA analysis results based on the average Occurrence (O), Detectability (D), Severity (S) and Risk Priority Number (RPN) scores. The values in brackets determine the minimum and maximum score across all raters (minimum, maximum).

**Subprocess**	**Failure Modes**	**Failure Causes**	**Failure Effects**	**O**	**D**	**S**	**RPN**	**Preventive Measures**
Gated Treatment	Irregular breathing cycle in the patient	Uncompliant patient	Determine the wrong exhalation/ inhalation. May underdose the target and overdose the OAR	4.7 (4,5)	6.3 (5,8)	7 (6,8)	207	Patient Education
CBCT Dose evaluation	Wrong CBCT/Plan image registered for CBCT Dose evaluation	Oversight of Physicist	Inaccurate dose evaluation on the CBCT	3.7 (3,5)	7.7 (6,9)	7.3 (7,8)	206	2nd Physicist Check
Gated Treatment	Error in calculating total RGPT shift during intra-fraction	Oversight of RT	Underdose to tumor and/or overdose to OAR	3.3 (2,4)	8 (7,9)	7.7 (7,8)	204	2nd RT Check
CBCT Bone match for patient alignment	Intra-fraction motion after CBCT	Bladder size increases or patients move intra-fraction	Underdose to tumor and/or overdose to OAR	4 (3,5)	9.3 (8,10)	5 (5,5)	187	RGPT Fluoroscopy
RGPT Marker match for patient alignment	RGPT marker match shifts exceed clinical protocols	Inaccurate patient setup/anatomy change/marker migration/ inconsistent breathing	Treated patients with exceeded RGPT shifts. May underdose the target and overdose the OAR	3 (3,3)	8 (7,8)	7.7 (7,8)	184	2nd RT Check / Patient Education
CBCT Bone match for patient alignment	Substantial contour change	Weight loss/gain or tumor growth/shrinkage	May underdose to tumor and/or overdose to OAR. Unable to proceed with treatment	5 (5,5)	6 (5,7)	5.7 (5,6)	170	Dose evaluation based on CBCT
RGPT Marker match for patient alignment	PIAS calculated an incorrect marker match shift	Machine fault	Underdose to tumor and/or overdose to OAR	2 (2,2)	9.3 (9,10)	8 (7,9)	149	Daily/Monthly RGPT QA
Physics Check	Wrong structure or ISO selected for RGPT shift calculation	Oversight of Physicist	The calculated RGPT shift may be more than the actual allowable RGPT shift. May underdose the target	2.7 (2,3)	7.7 (7,8)	7 (5,8)	143	2nd Physicist Check
Physics Check	Max allowable RGPT shift evaluated on the wrong structure	Oversight of Physicist	The calculated RGPT shift may be more than the actual allowable RGPT shift. May underdose the target	2.3 (2,3)	7.7 (7,8)	7.7 (7,9)	137	2nd Physicist Check
Physics Check	Created RGPT evaluation ISO different from Plan	Oversight of Physicist	The calculated RGPT shift may be more than the actual allowable RGPT shift. May underdose the target	2.3 (2,3)	7.7 (7,8)	7.7 (7,8)	137	2nd Physicist Check

**Table 2:** *Group 1 + 2* top ten FMEA analysis results based on the average Occurrence (O), Detectability (D), Severity (S) and Risk Priority Number (RPN) scores. The values in brackets determine the minimum and maximum score across all raters (minimum, maximum).

**Subprocess**	**Failure Modes**	**Failure Causes**	**Failure Effects**	**O**	**D**	**S**	**RPN**	**Preventive Measures**
Gated Treatment	Irregular breathing cycle in the patient	Uncompliant patient	Determine the wrong exhalation/ inhalation. May underdose the target and overdose the OAR	4.3 (3,5)	4.7 (3,8)	7 (5,8)	142	Patient Education
Gated Treatment	Patient treated without gating (moving tumor)	Oversight or Physicist/RT/Dosimetrist	Underdose to tumor and/or overdose to OAR	3.2 (2,4)	5 (3,10)	8.2 (7,10)	129	2nd Check by all parties
Gated Treatment	Error in calculating total RGPT shift during intra-fraction	Oversight of RT	Underdose to tumor and/or overdose to OAR	3 (1,4)	6 (3,9)	7 (4,9)	126	2nd RT Check
RGPT Marker match for patient alignment	RGPT marker match shifts exceed clinical protocols	Inaccurate patient setup/anatomy change/marker migration/ inconsistent breathing	Treated patients with exceeded RGPT shifts. May underdose the target and overdose the OAR	3.2 (1,4)	5.2 (1,9)	7.3 (4,10)	120	2nd RT Check / Patient Education
RGPT Marker match for patient alignment	Different marker is selected for matching and tracking	Oversight of RT	Underdose to tumor and/or overdose to OAR	3 (2,4)	4.7 (3,8)	7.7 (5,10)	107	2nd RT Check
CBCT Bone match for patient alignment	Intra-fraction motion after CBCT	Bladder size increases or patients move intra-fraction	Underdose to tumor and/or overdose to OAR	3.8 (3,5)	6 (1,10)	4.5 (3,5)	104	RGPT Fluoroscopy
RGPT Marker match for patient alignment	PIAS calculated an incorrect marker match shift	Machine fault	Underdose to tumor and/or overdose to OAR	1.7 (1,2)	8 (5,10)	7.5 (5,9)	100	Daily/Monthly RGPT QA
Gated Treatment	Gating tolerance is set larger than the protocol	Oversight of Physicist/RT	Underdose to tumor and/or overdose to OAR	3.7 (2,5)	4.5 (2,10)	5.8 (5,7)	96	2nd Physics/RT Check
CBCT Bone match for patient alignment	Substantial contour change	Weight loss/gain or tumor growth/shrinkage	May underdose to tumor and/or overdose to OAR. Unable to proceed with treatment.	5 (3,7)	3.7 (1,7)	5.2 (4,6)	95	Dose evaluation based on CBCT
CBCT Bone match for patient alignment	Applying marker match shift instead of bone match shift	Oversight of RT	May underdose to tumor and/or overdose to OAR	2.5 (2,3)	6.7 (5,10)	5.7 (4,7)	94	2nd RT Check

In addition, the analysis of the top ten failure modes revealed that all subprocess occurrences were relatively rare or unlikely (*O* ≤ 5). High RPN scores (>125) were primarily attributed to high severity (*S* ≥ 5) and/or high probability of undetected failures (*D* ≥ 5). There is a large difference between the individual highest RPN score as compared to the highest average RPN score. The individual RPN scores ranged from 1 to 320 for *Group 1* and 1 to 300 for *Group 1 + 2*. However, when averaged across all raters, RPN scores spanned from 2 to 207 for *Group 1* and 7 to 142 for *Group 1 + 2*.

## Discussion

This study presents the first comprehensive FMEA for the RGPT workflow, involving both medical physicists and radiation therapists. This is important as there are no established workflow and quality assurance (QA) protocols reported in the community and this analysis identified critical risk areas across the RGPT processes. Therefore, this study serves as a foundation for establishing a robust workflow and QA processes tailored to the specific demands of RGPT. Inter-rater agreement analysis was also done to highlight the potential differences in risk perception between the two disciplinary groups in radiation oncology.

## Average highest RPN for processes rated by *Group 1* and *Group 1 + 2*

The highest RPN value is observed in the *Re-CT and Replanning* process (RPN: 206), *Physics Plan Check* (RPN: 143), and *Treatment* process, particularly regarding irregular patient breathing cycles (RPN: 207 for *Group 1*, 142 for *Group 2*.

In the *Re-CT and Replanning* process, a dose evaluation based on the CBCT image is sometimes required to ensure that the correct dose is delivered to the target. One step of the dose evaluation is that the CBCT images are required to be registered to the original plan CT images. If the wrong images are registered, the evaluation will be invalid. This will lead to wrong information being given to the oncologist to make clinical decisions [33]. The average RPN value of 206 is mainly contributed by the high detectability (6 to 9) and severity (7 to 8) scores.

In the physics *Plan Check* process, the RGPT maximum allowable shifts are calculated. During this process, one of the steps is to create a new structure and isocenter. If the structure or isocenter is selected wrongly and the RGPT shifts are implemented during treatment, the wrong dose will be given to the target. This yields an average high RPN score of 143 with a high detectability and severity score of 7 to 8.

These high-risk processes necessitate immediate mitigation strategies. For instance, the high RPN in the Re-CT and Replanning process, stemming from potential errors in image registration, can be addressed by implementing cross-checks and comparing registrations with those done at the treatment machine. In the Physics Plan Check process, introducing a secondary review by another physicist could significantly reduce the risk associated with incorrect structure or isocenter selection.

Prior to the *Treatment* process, the *RGPT Feasibility Assessment* process yielded an average highest RPN score of 84. Similarly, this is related to the irregular breathing cycle of the patient. When a patient has an irregular breathing pattern, Patient selection and coaching can be performed before the assessment to reduce the occurrence of the failure mode [[33], [34], [35], [36]]. This is also the reason for a stringent assessment workflow as shown in Fig. 3 to prevent downstream high-risk failure modes.

In the *Treatment* process, the irregular breathing cycle of the patient can happen when the patient’s condition changes during treatment day or they become weaker after multiple treatment sessions. If the patient is treated at the wrong exhalation or inhalation phase, there will be underdosing to the target and overdosing to the OARs [37,38]. The high RPN score again reflects the need for proper patient education and coaching to mitigate the failure mode throughout the treatment and allow ample rest during the treatment session.

For the prostate treatment, generally, the target does not move as much as compared to the liver and lung region. There is also a bladder protocol [39,40] in place to ensure the reproducibility position of the target. Therefore the risk for RGPT treatment for prostate cancers is lower.

In addition, the top ten failure modes show that while the occurrence for all subprocesses is relatively low, the high RPN scores stem primarily from the severity of potential failures and challenges in detecting them. This pattern emphasizes the importance of detection mechanisms and preventive measures, even for rare events. The large difference between individual RPN scores and average RPN scores underscores the nature of risk assessment and highlights the need to standardize training and collaborative discussions to align perceptions across different disciplinary groups [41]. It suggests that relying solely on average RPN scores may obscure important individual insights, advocating for a more nuanced approach to risk evaluation in RGPT. With this FMEA analysis, preventive measures such as patient QA checks were established to mitigate these failure modes.

## Failure modes with high severity

Interestingly, our analysis revealed that some processes with high severity scores did not necessarily result in high RPNs. For example, using the wrong CT image phases during Treatment Planning could lead to a geographic miss of the target and overdosing the OAR might occur. This led to the highest average severity score (8.7). However, it has a relatively low RPN (46.2) due to existing multiple checks from the dosimetrist and the medical physicist.

From the results of *Group 1 + 2*, with an average severity score of 7 to 8, there are three sub-processes from the *Treatment* process – 1) A different marker was selected between marker matching and marker tracking, 2) An incorrect marker match shift was calculated by the imaging system and 3) Patient was treated without gating. The occurrence and detectability scores for (1) and (3) were low as there is a 2nd radiation therapist to check before the treatment starts. This leads to an average RPN score of 129.3 and 100 respectively. As for the machine fault where it shows the incorrect marker shifts, the average RPN score is 107.3. The average occurrence score is low (1.7) and this is due to our daily QA checks for the RGPT system. A plastic cube with ball bearings encased at known positions within the cube is used to ensure that the displayed shifts in the system match the known position of the ball bearings. This discrepancy underscores the importance of considering severity independently of RPN when prioritizing QA efforts.

## Inter-rater agreement

In this study, ICC was utilized to assess the degree of agreement among raters when evaluating various components of the FMEA of RGPT processes. This statistical measure provides information on the consistency of risk assessments across different disciplines and helps identify areas where perceptions of risk may vary [42]. Fig. 6 revealed higher intraclass correlations within *Group 1* compared to the combined *Group 1 + 2*. This disparity might be attributed to differences in understanding of QA processes between medical physicists and radiation therapists, as well as the limited clinical experience of only 15 patients treated with the RGPT system. Moreover, humans are poor at assessing risk [9,[43], [44], [45]] and thus, more time can be spent on explaining each failure mode to all raters.

A notable decrease in ICC for RPN, occurrence, and detectability in Processes 4 and 5 (*Treatment Planning* and *Physics Plan Check* respectively) can be seen. This decrease in agreement could be attributed to the different perceptions of individual medical physicists of what constitutes a potential issue. The discrepancies in evaluating occurrence and detectability could reflect differences in individual experiences with how often certain issues are encountered during treatment planning and plan checks.

With regards to the low ICC for Processes 2 and 6, both disciplinary groups have the same consensus of the failure modes with the highest severity and highest RPN score. The irregular breathing of the patient and the wrong phases selected for treatment planning were also deemed as the highest 20 % average RPN from TG290 concerning motion management for particle therapy [21].

These findings highlight the need for comprehensive training and explanation of failure modes across all disciplines involved in the RGPT workflow. Developing clear guidelines and fostering more frequent discussions among disciplines about potential risks in these processes could help improve consistency in risk assessment and ultimately enhance the safety and quality of RGPT treatments.

From a resource management perspective, our findings suggest that while individual assessments are logistically easier, they may lead to inconsistencies [46]. To balance efficiency and comprehensiveness, a hybrid approach can be performed where initial individual assessments are done followed by targeted group discussions for high-risk processes or areas of significant disagreement.

This should be complemented by regular reviews and updates of FMEA assessments to account for evolving practices and increased clinical experience with RGPT [8,17,26,47]. It is crucial to prioritize the development of standardized QA protocols that address the highest-risk failure modes identified in this study. These measures collectively will enhance the safety and efficacy of RGPT treatments while promoting a culture of continuous improvement and interdisciplinary cooperation.

## Conclusion

The first FMEA has provided crucial insights for developing comprehensive QA protocols and workflows. By addressing both high-severity processes and those with high RPN scores, we have established a foundation for the safe and effective implementation of RGPT. Additionally, as more centres adopt RGPT, we advocate for the establishment of a multi-institutional database to share experiences and refine best practices.

**Funding statement**

Hong Qi Tan is supported by the 10.13039/100016017Duke-NUS Oncology Academic Program Goh Foundation Proton Research Programme (08/FY2021/EX(SL)/92-A146), Clinical & Systems Innovation Support – Innovation Seed Grant (08/FY2022/P2/02-A68).

Wei Yang Calvin Koh is supported by the Ministry of Health, Singapore, MOH Health Innovation Fund (MH 110:12/12–30).

Author contribution

Study conception and design: Wei Yang Calvin Koh, Hong Qi Tan.

Data acquisition and analysis: Wei Yang Calvin Koh, Hong Qi Tan, Kah Seng Lew, Wan Ting Alice Kor, Nur Atiqah Binte Samsuri, Wei Siang Jason Chan.

Data interpretation: All authors.

Statistical analyses: Wei Yang Calvin Koh.

Obtained funding: Wei Yang Calvin Koh, Hong Qi Tan.

Administrative, technical, or material support: Wei Yang Calvin Koh, Hong Qi Tan.

Study supervision: Sung Yong Park.

Drafting of manuscript: Wei Yang Calvin Koh.

Approval of final manuscript: All authors.

## Declaration of competing interest

The authors declare that they have no known competing financial interests or personal relationships that could have appeared to influence the work reported in this paper.
